# Radiofrequency Ablation for the Treatment of Chronic Hip Pain: Does It Work?

**DOI:** 10.7759/cureus.117764

**Published:** 2026-10-11

**Authors:** Dimitrios I Gelalis, Dimitrios P Christakos, Ioannis Alatsathianos, Benav Bnavi, Hannah Katarina Rance, Ioannis Gkiatas, Vasilios Raoulis, Michael-Alexander Malahias

**Affiliations:** 1 Orthopaedic Surgery and Traumatology, Univeristy Hospital of Ioannina, Ioannina, GRC; 2 Orthopaedics and Traumatology, KAT General Hospital of Attica, Athens, GRC; 3 Epidemiology and Biostatistics, School of Medicine, European University Cyprus, Frankfurt, DEU; 4 Orthopaedics and Traumatology, School of Medicine, European University Cyprus, Frankfurt, DEU; 5 Orthopaedics and Traumatology, University Hospital of Ioannina, Ioannina, GRC; 6 Orthopaedics and Traumatology, University General Hospital of Larissa, Larissa, GRC

**Keywords:** chronic hip pain, minimally invasive therapy, osteoarthritis of the hip, radiofrequency ablation (rfa), radiofrequency thermocoagulation

## Abstract

Chronic hip pain is a common condition that can affect a person’s overall quality of life. Radiofrequency ablation (RFA) has gained attention as a minimally invasive technique that can reduce pain by targeting the small sensory nerves around the hip joint. While early reports are encouraging, clinical studies have shown mixed results. This meta-analysis aimed to clarify the efficacy of RFA in the management of chronic hip pain. A systematic review and meta-analysis were conducted using PubMed/Medline, Scopus, and Cochrane databases from January 2007 to June 2025 to identify studies reporting clinical outcomes of hip RFA. Standardized mean differences (Hedges’ g) and pooled percentage changes in pain were calculated using a random-effects model. Sensitivity, subgroup, and interaction analyses assessed the influence of RFA type, imaging guidance, and study design. Across 449 patients, RFA produced significant pain reduction at all time points: 61% at early-term (one month) (39.09%, 83.06%), 58% at short-term (three months) (34.85, 81.12), 45% at mid-term (six months) (30.24%, 59.56%), and 35% at long-term (nine months) (19.29%, 50.79%). Effect sizes were largest within the first three months (g=5.9443 (3.2384, 8.6501)). Subgroup analyses revealed no significant differences among RFA types, whereas combined ultrasound-fluoroscopy guidance tended to yield better outcomes than either guidance modality alone. This review demonstrates that RFA is an effective and safe minimally invasive therapy for management of chronic hip pain, offering substantial pain reduction in the early- and short-term periods.

## Introduction and background

One of the most prevalent causes of hip pain is osteoarthritis (OA), a progressive and multifaceted disease that affects over half a billion people globally as of 2019 [1]. OA is characterized by the structural deterioration of joint cartilage, leading to symptoms such as joint pain, stiffness, and impaired function [2]. Hip osteoarthritis (HOA) specifically involves the degeneration of the articular cartilage in the hip joint, resulting in joint space narrowing, osteophyte formation, subchondral cysts, and sclerosis [3]. Other hip-related conditions, including avascular necrosis (AVN) and post-surgical complications, can also contribute to chronic pain, progressive joint degeneration, and reduced mobility [4].

Current management strategies for mild to moderate forms of HOA include exercise, weight loss, and patient education, with pharmacological treatments such as nonsteroidal anti-inflammatory drugs (NSAIDs), steroid injections, prolotherapy (hyaluronic acid injections), autologous platelet-rich plasma (PRP) injections, and bone marrow concentrate (BMAC) infiltration providing short- to mid-term pain relief [5]. In severe HOA cases with chronic intractable pain, total hip replacement (THR) is the gold standard treatment [6]. However, total hip arthroplasty is a surgical operation that carries a small but important risk of complications ranging from wound complications and implant loosening to reoperation or even death [7].

Moreover, middle-aged to elderly patients are the typical THR population that often present with significant comorbidities that increase perioperative risk and affect recovery. Nearly one-third of THR candidates fall into moderate to high comorbidity status categories, in which severe comorbidities can lead to denial of surgery [8]. As the proportion of the elderly population continues to rise, octogenarians and older individuals account for approximately 5-8% of primary THR recipients and are associated with increased perioperative vulnerability [9]. In our experience, a noteworthy subgroup of patients is declining elective surgery and prefers non-surgical management mainly due to fear of complications, fear of anesthesia, fear of postoperative pain, or even lack of a social environment to support the rehabilitation process, which varies between 6-12 weeks.

As OA prevalence continues to rise due to aging populations and lifestyle factors, there is an increasing demand for alternative, minimally invasive interventions that can provide effective and sustained pain relief while preserving joint function [1]. One such emerging technique is radiofrequency ablation (RFA), a minimally invasive interventional procedure that has shown promising results in managing chronic joint pain, particularly in patients who do not respond to conservative treatments or are unsuitable for surgery [4]. Initially used for spinal and oncologic pain management, RFA's application in joint-related conditions, particularly hip pathology, remains an evolving area of study [10]. Multiple RFA techniques have emerged, including conventional (thermal), cooled, and pulsed RFA, each with distinct mechanisms of action and clinical applications [10]. RFA was first used in the 1970s for the management of back pain and introduced in 2008 for chronic knee pain [11]. RFA of the hip joint delivers controlled high-frequency electrical currents to selectively ablate nociceptive pathways responsible for pain transmission, specifically targeting the articular branches of the obturator and femoral nerves (L2-L4) [12].

Despite its growing use, existing literature presents heterogeneous results regarding hip RFA's efficacy and long-term outcomes. Some studies report significant pain reduction and functional improvement, while others highlight inconsistent benefits and the potential for nerve regeneration leading to recurrent pain [13]. Additionally, questions remain regarding the impact of imaging modality (ultrasound, fluoroscopy, or combined techniques), RFA type, and study design on treatment outcomes.

To address these gaps in the literature, this study aimed to evaluate the clinical outcomes of RFA for hip pain by analyzing the effect size difference in pain and function across different time points: early-term (one month), short-term (three months), mid-term (six months), and long-term (nine months). Specifically, the primary aim of this study was to evaluate the magnitude of pain reduction following RFA across different time points. Secondary outcomes included the impact of the imaging modality used for RFA guidance (ultrasound, fluoroscopy, or combined fluoroscopy-ultrasound) and the type of RFA (thermal, cooled, or pulsed) on pain relief and functional improvement, as well as the association between study design (prospective vs. retrospective) and reported efficacy and safety outcomes. By addressing these topics, this study seeks to contribute to a better understanding of RFA’s role in clinical practice, guiding future research and optimizing patient selection criteria for this minimally invasive intervention. Our primary hypothesis was that hip RFA treatment improves clinical outcomes by significantly reducing hip pain in the early-, short-, mid-, and long-term periods.

## Review

Methodology

Search Criteria

This research was conducted in accordance with the Preferred Reporting Items for Systematic Reviews and Meta-Analyses (PRISMA) guidelines [14]. The US National Library of Medicine (PubMed/MEDLINE), Scopus, and the Cochrane Database of Systematic Reviews were queried for publications utilizing the following search string: ("radiofrequency ablation" OR "radiofrequency denervation" OR "pulsed radiofrequency" OR "cooled radiofrequency" OR "conventional radiofrequency" OR thermocoagulation) AND ("hip pain" OR "chronic hip pain"), from 01/01/2007 to 01/06/2025. As RFA is a continuously evolving and improving technique, which may influence clinical outcomes and thereby affect the results of a systematic review, studies published prior to 2007 were excluded from the present meta-analysis. Two authors independently conducted all the searches and screened the titles and abstracts to identify relevant studies. Only abstracts that reported clinical outcomes for patients treated with RFA using validated measures were included. If the title and abstract of each study contained insufficient information, the full manuscript was reviewed. An additional search was conducted by screening the reference list of each selected article. When studies reported outcomes on the same cohort of patients, the one with the longest follow-up was selected. If there was disagreement between the two reviewers, discrepancies were resolved by discussion.

Study Selection and Data Extraction

Studies were included if they reported outcomes for pain intensity and/or functional capacity using validated measures after RFA for hip pain. Only papers published in the English language were included in the study. Studies that were still ongoing were excluded. Case reports were also excluded. The quality assessment of the studies for methodological deficiencies, as a common alternative to risk of bias, was examined by the Modified Coleman Methodology Score [15]. To ensure methodological transparency and compliance with reporting standards, we performed a risk of bias evaluation using the Risk of Bias in Non-randomized Studies of Interventions (ROBINS-I) tool for non-randomized studies [16]. The level of evidence in the included studies was determined using the Oxford Centre for Evidence-Based Medicine Level of Evidence (LoE) [17]. Pain intensity was assessed using the Numeric Rating Scale (NRS), Visual Analog Scale (VAS), and other standardized pain scores. Functional capacity was measured with tools such as the Western Ontario and McMaster Universities Osteoarthritis Index (WOMAC) [18] and the Hip Disability and Osteoarthritis Outcome Score (HOOS) [19].

The two reviewers independently extracted the data, including the title, year of publication, authors, study design, number of patients, pain score, functional outcome score, patient-reported outcome measures (PROMs), and complications.

Data Preparation and Composite Scores

The treatment groups were defined as RFA therapy for hip pain and control interventions involving any conservative treatment for hip pain (including pain medications, NSAIDs, corticosteroid injections, physiotherapy), with the control group explicitly excluding any type of RFA. Results were stratified into four categories based on the time points at which the effects of RFA therapy were measured in the studies included in this review. Thus, the early-term period refers to results measured up to four weeks after treatment, the short-term period up to 12 weeks, the mid-term period up to 24 weeks, and the long-term period up to 36 weeks or more. For studies that did not explicitly report mean values and standard deviations (SDs), the WebPlotDigitizer tool [20] was utilized to extract these metrics from graphical plots.

The standardized mean difference (SMD) was computed for pre- and post-intervention scores, assuming a correlation coefficient of 0.5 between baseline and post-intervention variances. To standardize outcomes across studies, composite scores were created for studies reporting multiple pain outcomes. For studies reporting the same outcome at the same time point, composite effect sizes were calculated. Composite scores were derived using the following formula:

$\text{Composite Hedges'}\ g = \frac{\sum g_i}{n}$

where g_i_ represents the individual effect sizes, and n is the number of studies.

The composite variance (V_g_) was calculated as follows:

$V_g = \frac{1}{n^2} \left( \sum V_{g_i} + 2 \sum_{i&lt;j} r \sqrt{V_{g_i}} \sqrt{V_{g_j}} \right)$

where r = 0.5 is the assumed correlation coefficient between studies.

Meta-analysis

A random-effects meta-analysis was performed using the metafor package in R (version 4.4.1; R Foundation for Statistical Computing, Vienna, Austria) to pool effect sizes across studies. Hedges’ g was used as the effect size metric, with variance estimates derived from study-specific SDs and sample sizes. Percentage changes in pain scores were estimated by approximately converting the standardized effect sizes using baseline descriptive statistics. Specifically, the estimated percentage change was calculated as follows:

$\text{Percentage Change} = g \times \left( \frac{\text{Baseline SD}}{\text{Baseline Mean}} \right) \times 100$

where g is the Hedges' g effect size.

These percentages represent derived estimates rather than directly pooled observed percentage reductions.

Heterogeneity was assessed using the I^2^ statistic, with values >50% indicating substantial heterogeneity.

Sensitivity Analyses

Primary sensitivity analysis: A leave-one-out analysis was conducted to evaluate the robustness of the results. This involved iteratively removing one study at a time and recalculating the pooled effect size to assess whether any single study disproportionately influenced the overall results.

Secondary sensitivity analysis: A secondary sensitivity analysis was performed by excluding studies where subjects did not undergo a diagnostic block prior to the operation to confirm their suitability for RFA or where the severity of their condition was not objectively measured using established grading systems (e.g., Tonnis grading for HOA or Kellgren-Lawrence Grade for knee OA) [21,22]. This analysis aimed to evaluate the impact of including studies with less stringent patient selection criteria on the overall results.

Subgroup and Interaction Analyses

Subgroup analyses were performed to explore potential sources of heterogeneity. We compared outcomes based on the imaging modality used for RFA guidance (e.g., ultrasound, fluoroscopy, and fluoroscopy-ultrasound combined). A second comparison was made between studies with different designs (prospective or retrospective studies). A third comparison was made between the outcomes of different types of RFA (e.g., thermal, cooled, pulsed).

Forest Plots and Percentage Change

Forest plots were generated to visualize effect sizes and 95% confidence intervals (CIs) for each time point (early-term, short-term, mid-term, long-term) for both pain and functional outcomes.

Statistical Analyses

All analyses were conducted in R (version 4.4.1) using the metafor and dplyr packages. The significance level was set at p<0.05.

Results

The literature search initially yielded 181 relevant citations (Figure 1). After removal of duplicate studies, 138 were subject to application of the predetermined inclusion and exclusion criteria. Following the application of these criteria, 61 studies were subject to a full-text screening process. Ultimately, 12 studies were included for analysis (Table 1) [12,23-33].

**Figure 1:**
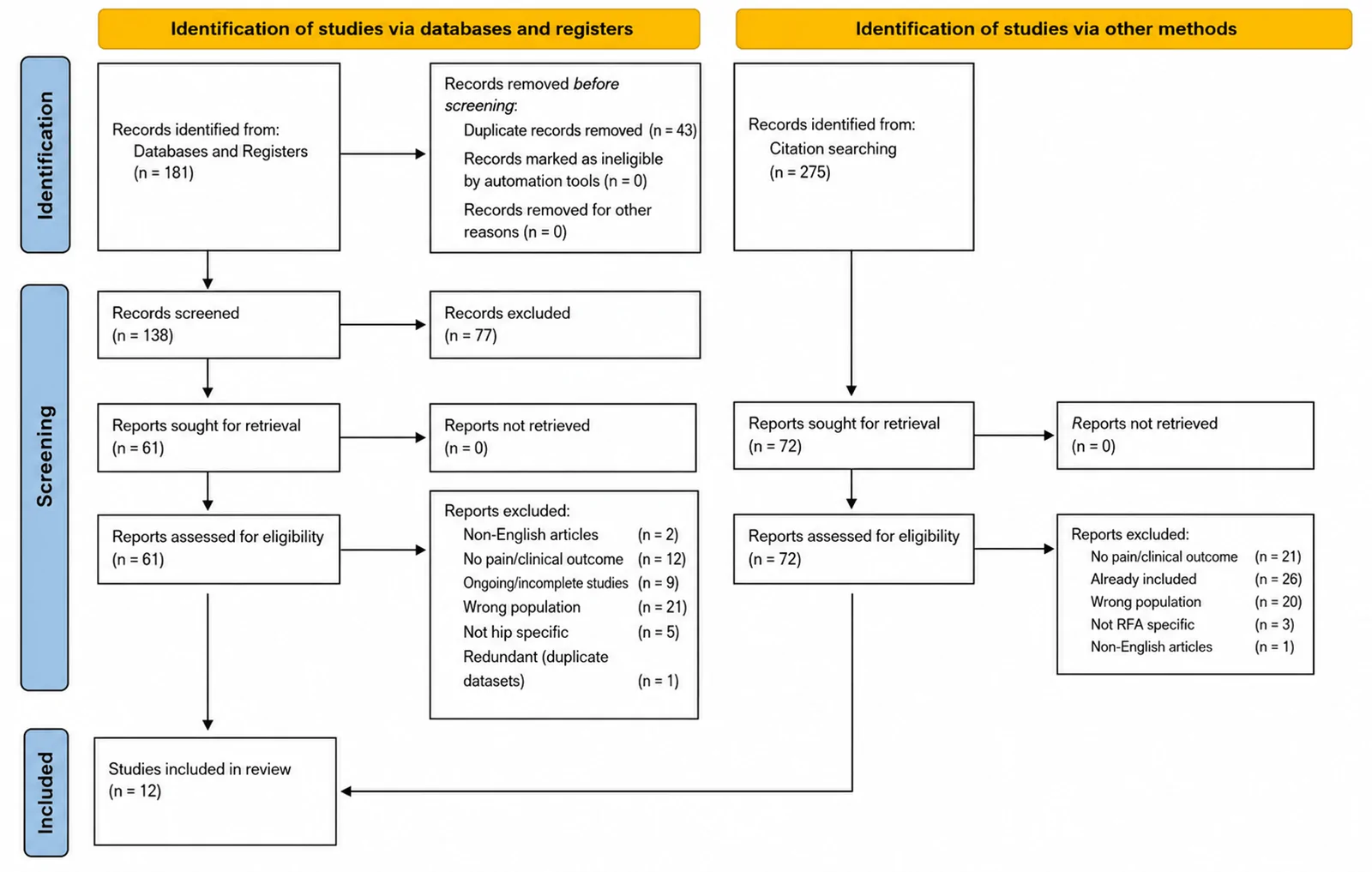
PRISMA flow diagram demonstrating the systematic review process. PRISMA: Preferred Reporting Items for Systematic Reviews and Meta-Analyses

**Table 1:** Studies included in the meta-analysis. OA: osteoarthritis; M: male; F: female; postop: postoperative, AVN: avascular necrosis

Author (year)	Type of study	Modified Coleman Score	Number of patients	Mean age	Sex	Reason for surgery	RFA type	Level of evidence
Tinnirelo et al. (2018) [12]	Retrospective observational study	50	14	67.11	10F, 4M	OA (Kellgren-Lawrence II-IV)	Pulsed	IV
Tran et al. (2022) [23]	Prospective observational study	52	11	61.4	3F, 8M	OA (Tonnis II-III)	Cooled	ΙV
Kapural et al. (2021) [24]	Retrospective observational study	55	84	57	63F, 21M	Chronic hip pain	Cooled	IV
Petroni et al. (2024) [25]	Retrospective observational study	51	44	71.6	26F, 18M	Chronic hip pain	Thermal	IV
Chye et al. (2015) [26]	Prospective non-randomized study	64	29	65.53	19F, 10M	OA	Pulsed	III
Vanaclocha et al. (2016) [27]	Retrospective observational study	62	91	71.91	52F, 39M	OA (Tonnis I-II)	Thermal	IV
Rivera et al. (2012) [28]	Prospective non-randomized study	47	18	-	-	OA (Tonnis I-II)	Thermal	III
Naber et al. (2019) [29]	Prospective observational study	45	7	56	4F, 3M	Chronic hip pain (due to AVN)	Cooled	IV
Diwan et al. (2024) [30]	Retrospective observational study	56	31	66.6 ± 5.7	18F, 13M	Chronic hip pain	Pulsed	IV
Karaoglu et al. (2024) [31]	Prospective observational study	51	48	60.7	29F, 19M	OA 77.1% / postop hip pain 12.5% / malignancy 8.3% / AVN 2.1%	Thermal	IV
Correia et al. (2024) [32]	Retrospective observational study	54	26	63	11F, 15M	OA (Tonnis II-III)	Thermal	IV
Fishchenko et al. (2021) [33]	Prospective observational study	54	46	64.5 ± 1.2	-	OA (Kellgren-Lawrence III-IV)	Thermal	IV

Characteristics of Included Studies

Six out of 12 studies (50%) were retrospective observational studies with a level IV LoE [12,24,25,27,30,32]. Four out of 12 studies (33%) were prospective observational studies with a level IV LoE [23,29,31,33], and two out of 12 (17%) were prospective non-randomized clinical trials with a level III LoE [26,28]. The mean Modified Coleman Score was 53.4 (out of 100), with scores ranging from 45 to 64 (out of 100), indicating a moderate methodological quality. Risk of bias was assessed using the ROBINS-I tool across all studies. All studies were classified as having a serious risk of bias, mainly attributable to the high risk of bias arising from confounding. The results of the risk assessment are presented in Table 2. A total of 449 patients were included in the studies.

**Table 2:** Risk of bias assessed using the ROBINS-I tool. ROBINS-I: Risk of Bias in Non-randomized Studies of Interventions

Study	Bias due to confounding	Bias in the selection of participants	Bias in the classification of interventions	Bias due to deviations from intended interventions	Bias in the measurement of outcomes	Bias in the selection of the reported result	Overall risk of bias
Tinnirelo et al. (2018) [12]	Serious	Moderate	Low	Low	Moderate	Moderate	Serious
Tran et al. (2022) [23]	Serious	Moderate	Low	Low	Moderate	Low	Serious
Kapural et al. (2021) [24]	Serious	Moderate	Low	Low	Moderate	Moderate	Serious
Petroni et al. (2024) [25]	Serious	Moderate	Low	Low	Moderate	Moderate	Serious
Chye et al. (2015) [26]	Serious	Moderate	Low	Low	Moderate	Low	Serious
Vanaclocha et al. (2016) [27]	Serious	Moderate	Low	Low	Moderate	Moderate	Serious
Rivera et al. (2012) [28]	Serious	Moderate	Low	Low	Moderate	Low	Serious
Naber et al. (2019) [29]	Serious	Moderate	Low	Low	Moderate	Low	Serious
Diwan et al. (2024) [30]	Serious	Moderate	Low	Low	Moderate	Moderate	Serious
Karaoglu et al. (2024) [31]	Serious	Moderate	Low	Low	Moderate	Low	Serious
Correia et al. (2024) [32]	Serious	Moderate	Low	Low	Moderate	Moderate	Serious
Fishchenko et al. (2021) [33]	Serious	Moderate	Low	Low	Moderate	Low	Serious

Pain Outcomes

The random effects demonstrated significant reductions in pain following RFA, with clinically meaningful improvements observed across all time points (Table 3, Figure 2).

**Table 3:** Meta-analysis results for pain reduction following RFA across time points. Main analysis included all eligible studies. Restriction analysis excluded studies without diagnostic block confirmation or objective severity grading. Subgroup analyses were performed only when sufficient data were available (≥2 categories with ≥2 studies each). Significant subgroup differences are displayed with effect estimates and 95% CIs; NS indicates no significant difference between subgroups. For main analysis: Early-term, short-term, and mid-term included all three guidance categories (fluoroscopy, ultrasound, combined) and all RFA types (thermal, cooled, pulsed); long-term missed cooled as an RFA type. For restriction analysis: Category availability varied by time point due to study exclusions. Only comparisons meeting feasibility criteria are reported. NS: not significant (p≥0.05); Comb = combined (fluoroscopy and ultrasound); Prosp: prospective study design; NA: not applicable (insufficient data for subgroup comparison, requiring ≥2 categories with ≥2 studies each); RFA: radiofrequency ablation; CI: confidence interval *p<0.1 indicates marginally significant findings.

Time point	Main analysis g (95% CI)	Pooled % change (95% CI)	I²	Sensitivity	Subgroup: guidance	Subgroup: RFA type	Subgroup: design
Main analysis							
Early-term	5.94 (3.24, 8.65)	61.08 (39.09, 83.06)	98.15%	Consistent	Comb: g=6.06 (1.61, 10.51)	NS (p=0.38)	Prosp: g=7.40 (1.70, 13.10)
Short-term	4.87 (2.19, 7.55)	57.99 (34.85, 81.12)	98.33%	Consistent	Comb: g=5.52 (0.13, 10.92)	NS (p=0.22)	Prosp: g=5.53 (0.07, 10.98)
Mid-term	4.05 (2.19, 5.91)	44.90 (30.24, 59.56)	98.68%	Consistent	Comb: g=4.80 (0.84, 8.75)	NS (p=0.41)	Prosp: g=4.84 (1.59, 8.10)
Long-term	3.53 (0.37, 6.69)	35.04 (19.29, 50.79)	99.51%	Variable	Comb*: g=5.67 (-0.60, 11.94)	NA	NA
Restriction analysis							
Early-term	5.50 (1.80, 9.21)	50.42 (27.93, 72.90)	98.33%	Consistent	Comb (vs Fluoro): g=7.91 (1.94, 13.88)	p=0.06	NA
Short-term	4.23 (0.63, 7.83)	51.24 (24.74, 77.74)	98.25%	Variable	NA	NS (p=0.30)	NA
Mid-term	4.18 (1.69, 6.67)	44.61 (27.42, 61.80)	98.90%	Consistent	Comb: g=6.73 (1.20, 12.26)	NS (p=0.39)	Prosp: g=6.15 (2.08, 10.22)
Long-term	3.53 (0.37, 6.69)	35.04 (19.29, 50.79)	99.51%	Variable	Comb*: g=5.67 (-0.60, 11.94)	NA	NA

**Figure 2:**
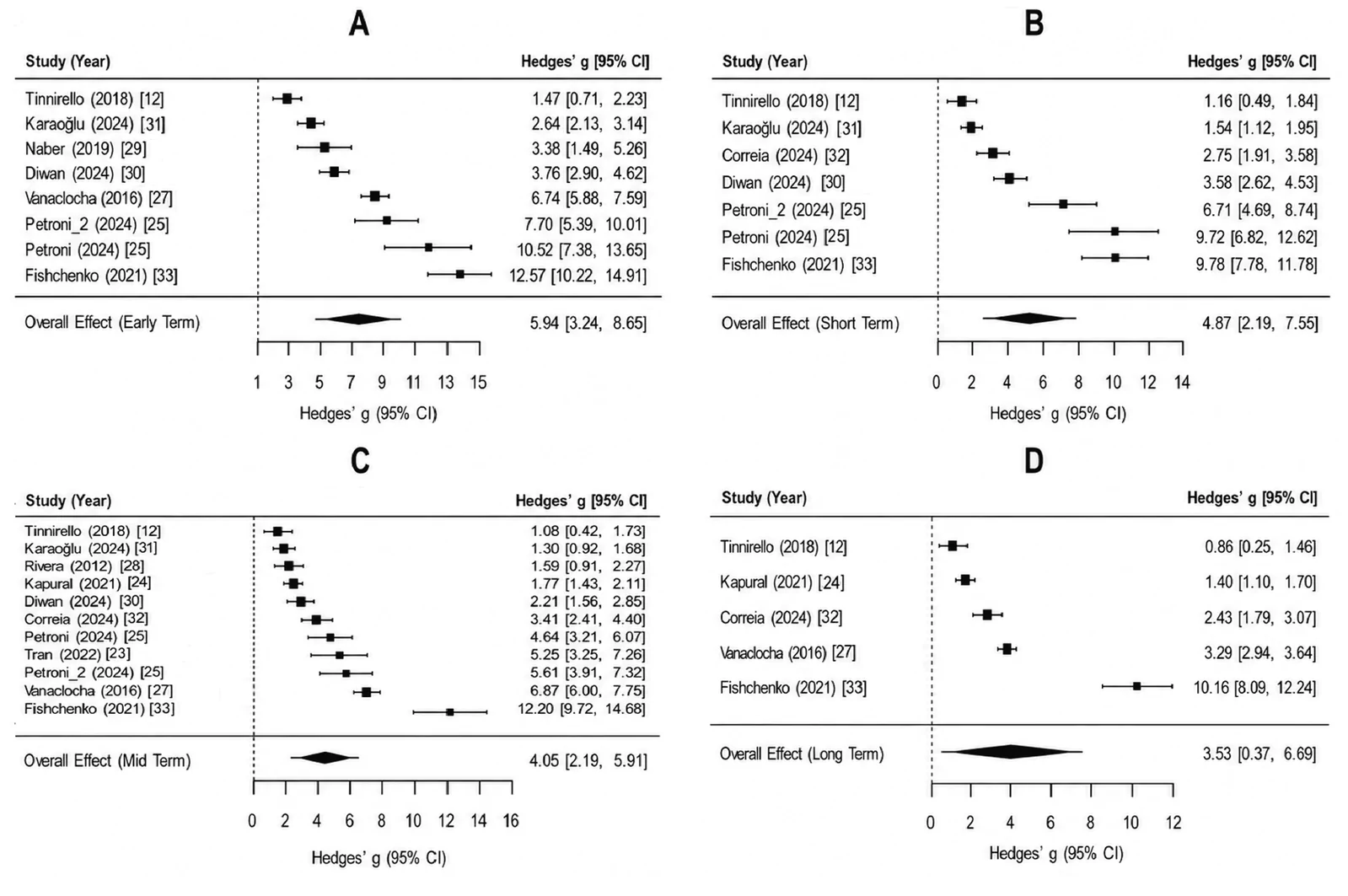
Forest plot of pain outcomes following radiofrequency ablation across time points: early-term (A), short-term (B), mid-term (C), and long-term (D). CI: confidence interval

Primary Outcome

The percentage change in pain scores from baseline, along with the corresponding effect sizes (Hedges' g), was as follows: Early-term pain scores decreased by 61.08% (39.09%, 83.06%), with a large effect size of g=5.9443 (3.2384, 8.6501). Short-term pain scores improved by 57.99% (34.86%, 81.12%), corresponding to a substantial effect size of g=4.8706 (2.1885, 7.5528). In the mid-term, pain reduction was 44.89% (30.23%, 59.56%), with a moderate to large effect size of g=4.0498 (2.1913, 5.9083). Long-term pain scores decreased by 35.04% (19.29%, 50.79%), reflecting a moderate effect size of g=3.5292 (0.3668, 6.6917).

Heterogeneity

Substantial heterogeneity was observed across all time points, with I^2^ values ranging from 98.15% (early-term) to 99.51% (long-term). This suggests significant variability in study populations, methodologies, or RFA protocols, which should be considered when interpreting the results.

Sensitivity Analyses

Primary sensitivity analysis (leave-one-out): The results were generally consistent across time points, with no single study disproportionately affecting the pooled percentage changes or effect sizes, except at the long-term time point.

Secondary sensitivity analysis (exclusion of studies without diagnostic block or objective severity grading): When studies without a prior diagnostic block or objective severity grading (e.g., Tonnis or Kellgren-Lawrence Grade) were excluded, the percentage changes remained consistent, suggesting that the inclusion of studies with less stringent patient selection criteria may not influence the outcomes.

Subgroup Analyses

It is worth examining the differences in guidance methods used throughout the papers. The methods of choice were ultrasound, fluoroscopy, or, in some cases, a combination of the aforementioned. Subgroup analysis by guidance method revealed a consistent advantage for a combination of ultrasound and fluoroscopy. However, in the secondary sensitivity analysis for short-term outcomes, significant differences were observed (p=0.0036) for all guidance methods. One of the subgroup analyses based on the RFA types (thermal, cooled, pulsed) showed no significant differences between the RFA types at any time point (p>0.05). The study designs in this review varied. The outcomes of the prospective studies consistently demonstrated larger percentage changes compared to retrospective studies, with significant differences observed for both study designs in the long-term (p<0.0001) and in the secondary sensitivity analysis, except for the mid-term.

Discussion

Our meta-analysis shows that patients treated with hip RFA experienced improved outcomes in terms of pain reduction across all time periods. This improvement was independent of the type of RFA method that was utilized.

Another meta-analysis of six studies by Ciaffi et al., published in 2024, presents results consistent with our study's findings [34]. In this meta-analysis, the improvement in pain reduction was apparent from the early term and was sustained until 12 months. Only three of the studies included in this meta-analysis were also part of our meta-analysis.

All types of RFA showed a crucial improvement in pain according to VAS or NRS scores. Tran et al. noted in a prospective study that patients receiving cooled RFA (C-RFA) showed improvement in HOOS scores at six months [23]. A retrospective study by Kapural et al. confirmed these results, with over half of the patients reporting more than 50% pain relief using C-RFA for up to 12 months [24]. Petroni et al. documented the use of thermal RFA with different approaches, reporting notable improvement in NRS scores [25]. Tinnirello et al. found that 57% of the patients showed pain improvement of more than 50% at six months [12]. Chye et al. reported the superiority of pulsed radiofrequency (PRF) over conservative treatment based on pain scores [26]. Diwan et al. mentioned significant pain recovery up to six months post-PRF without documenting any adverse effects [30].

Additionally, it is well noted that in patients with advanced OA, the use of RFA is rather helpful. In a prospective study, Fishchenko et al., after performing RFA in patients with OA grade 3-4, reported VAS score reductions in 69.5% of patients at six months, while around 50% maintained this relief status at 12 months [33]. Particularly promising, though not supported by other studies in the literature, is the fact that the effect of RFA on pain reduction in OA patients can last up to two years post-RFA, as suggested by a prospective observational study by Vanaclocha et al. [27].

As other studies show, RFA could be proven as an effective alternative for patients with severe contraindications to surgery or even those suffering from chronic hip pain due to other reasons except HOA, such as AVN. Rivera et al., in a prospective cohort study focusing on patients contraindicated for surgery, reported improved Harris Hip Score (HHS) and decreased VAS and WOMAC scores at six months [28]. Naber et al. studied patients with AVN and found >50% pain relief sustained between 70 and 250 days using C-RFA [29].

Another consideration is the approach used in performing RFA. Studies have shown that the approach used to perform RFA can influence outcomes by targeting as much of the joint’s sensory innervation as possible. Petroni et al., in a retrospective study, suggested that a combined anterior-posterior approach compared with anterior-only ablation yields better outcomes in terms of pain reduction at six months [25].

Moreover, although RFA is generally considered a safe technique with infrequent complications, the literature has reported specific adverse events, mainly related to injuries of vital structures located in the vicinity of the target articular nerves (femoral artery, femoral vein, distal motor branches of the obturator nerve (ON), femoral nerve (FN), and sciatic nerve (SN)). A systematic review by Cheney et al. found that the most common complications were transient paresthesias, while the most severe included quadriceps paralysis/femoral neuropathy. Other complications included arterial puncture/hematomas, which usually resolved with compressive dressing [13]. Kapural et al. and Diwan et al., in their respective studies, underline the importance of an ultrasound-guided approach for RFA as it is believed to minimize the complication risks [24,30].

Last but not least, it is important to evaluate the use of RFA with respect to symptom and pain relief. As indicated in our findings, the greatest pain reduction occurs in the early- and short-term periods after the procedure. Beyond six months, the effect tends to diminish, so performing a repeat procedure may provide additional pain relief to patients and help maintain positive outcomes for an even longer period. Repeating RFA could be a viable practice, as it does not require a significant recovery period and its results are noticeable from the very same day. Ηowever, its effectiveness and safety require further investigation in studies specifically evaluating repeat procedures. We should also bear in mind that targeting as many articular nerves as possible during RFA can help achieve the best possible outcomes [31-33]. 

Limitations

Limitations of this study include those inherent in the approach to conducting a systematic review and meta-analysis. Due to our strict inclusion and exclusion criteria, only 12 studies were selected for evaluation. Not all studies reported the same data points, and there were studies missing one or more of the variables we included in our analysis. As a result, there was variability in the overall pool of patients evaluated for each outcome. This variation in the total number of patients assessed for each outcome must be considered when interpreting these results. Furthermore, all included studies were assessed as having a serious risk of bias, and the evidence was predominantly observational, with no randomized controlled trials, which limits causal interpretation. The absence of randomized controlled trials prevents us from generalizing the results to the entire population and restricts the application of RFA only to patients in whom all other treatment options have failed. Additionally, the absence of comparative studies regarding the guidance method used in RFA limits our ability to determine which guidance technique provides the most reliable outcomes. The sample size was small, reducing the statistical power and increasing susceptibility to effect overestimation. Formal funnel-plot asymmetry testing was not performed because most pooled analyses included fewer than 10 studies; therefore, publication bias and small-study effects cannot be excluded. More clinical studies are needed to establish the most appropriate method for achieving the best outcomes in general and specifically within each target group.

## Conclusions

According to this review, hip RFA appears to be an effective, relatively safe, minimally invasive therapy for the management of chronic hip pain, offering pain reduction in the early- and short-term periods, as well as meaningful benefits in the mid- and long-term follow-up. Outcomes varied across studies due to differences in technique, imaging, and study designs. The main evidence supports RFA as a valuable alternative for patients who are not candidates for surgery. An important parameter is the low rate of complications, along with the fact that repeated treatment may help sustain pain relief beyond six months. Further studies are needed to define the different aspects for achieving the best outcomes in this procedure, based on procedural approaches, imaging guidance, and the understanding of long-term durability.
